# Genome-Based Approach Delivers Vaccine Candidates Against *Pseudomonas aeruginosa*

**DOI:** 10.3389/fimmu.2018.03021

**Published:** 2019-01-09

**Authors:** Irene Bianconi, Beatriz Alcalá-Franco, Maria Scarselli, Mattia Dalsass, Scilla Buccato, Annalisa Colaprico, Sara Marchi, Vega Masignani, Alessandra Bragonzi

**Affiliations:** ^1^Infection and Cystic Fibrosis Unit, Division of Immunology, Transplantation and Infectious Diseases, IRCCS San Raffaele Scientific Institute, Milan, Italy; ^2^GSK, Siena, Italy; ^3^Dipartimento di Scienze Cliniche e Biologiche, Universitá degli Studi di Torino, Turin, Italy

**Keywords:** *Pseudomonas aeruginosa*, reverse vaccinology, vaccine, respiratory infection, mouse model

## Abstract

High incidence, severity and increasing antibiotic resistance characterize *Pseudomonas aeruginosa* infections, highlighting the need for new therapeutic options. Vaccination strategies to prevent or limit *P. aeruginosa* infections represent a rational approach to positively impact the clinical outcome of risk patients; nevertheless this bacterium remains a challenging vaccine target. To identify novel vaccine candidates, we started from the genome sequence analysis of the *P. aeruginosa* reference strain PAO1 exploring the reverse vaccinology approach integrated with additional bioinformatic tools. The bioinformatic approaches resulted in the selection of 52 potential antigens. These vaccine candidates were conserved in *P. aeruginosa* genomes from different origin and among strains isolated longitudinally from cystic fibrosis patients. To assess the immune-protection of single or antigens combination against *P. aeruginosa* infection, a vaccination protocol was established in murine model of acute respiratory infection. Combinations of selected candidates, rather than single antigens, effectively controlled *P. aeruginosa* infection in the *in vivo* model of murine pneumonia. Five combinations were capable of significantly increase survival rate among challenged mice and all included PA5340, a hypothetical protein exclusively present in *P. aeruginosa*. PA5340 combined with PA3526-MotY gave the maximum protection. Both proteins were surface exposed by immunofluorescence and triggered a specific immune response. Combination of these two protein antigens could represent a potential vaccine to prevent *P. aeruginosa* infection.

## Introduction

*P. aeruginosa* infections are among the most severe public health issues. This opportunistic bacterium belongs to the multi-drug resistant (MDR) ESKAPE pathogens, along with *Enterococcus faecium, Staphylococcus aureus, Klebsiella pneumoniae, Acinetobacter baumannii*, and *Enterobacter*. According to data from Centers for Disease Control, *P. aeruginosa* is responsible for millions of infections each year in the community, 10–15% of all healthcare-associated infections, with more than 300,000 cases annually in the EU, USA and Japan (1). Patients hospitalized in intensive care units (ICU) run a high risk of acquiring *P. aeruginosa* as they may develop ventilator-associated pneumonia (VAP) and sepsis (2–4). Other risk groups are patients with a compromised immune system, either from immunosuppressive therapies and underlying diseases such as cancer, AIDS or hereditary cystic fibrosis (CF). This high prevalence is partly due to the vast arsenal of virulence factors that facilitates acute infections and the propensity of *P. aeruginosa* to form highly structured biofilm communities that cause chronic infections (5).

Taking the severity of the illness into account, current treatment guidelines for the management of bacterial infection recommend single antibiotic or combination therapy. Despite the wide arsenal of drugs for *P. aeruginosa* infections available on the market, inefficacy of these treatments is commonplace. Resistance rapidly emerges, usually linked to intrinsic bacterial resistance mechanisms, development of new antibiotic resistance, and/or limited penetration of antibiotics into biofilms (3, 6). Development of antibiotics with a novel mode of action and/or alternative therapies remains an urgent need for patients. Antibacterial agents launched in recent decades were modifications of existing molecules; the development of entirely new classes of antibiotics has been largely abandoned (7). Immunotherapy for preventing pulmonary infection has also been tested (8), but clinical efficacy has been disappointing (9, 10). Immunization strategies do not cover *P. aeruginosa* infection in healthcare practices.

In recent years, remarkable progress has been made in the identification of *P. aeruginosa* virulence factors and their variations among different infection processes. It has been more accurately recognized that *P. aeruginosa* is an antigenically variable microorganism that adapts easily to different growth conditions and escapes host immune recognition. The high variability of the proteins among different *P. aeruginosa* strains and within the same strain, grown in diverse environmental conditions, may represent a serious obstacle to the development of a globally effective anti–*P. aeruginosa* vaccine (10). So far, *P. aeruginosa* vaccine candidates have been found by classical approach—by identifying more abundant surface proteins and oligosaccharides or by selecting specific virulence factors, according to their relevance in the disease outcome. Integrated genomics and proteomics approaches have been recently used to predict vaccine candidates against *P. aeruginosa* (11). Although several vaccine formulations have been tested clinically, none has been licensed (10, 12). *P. aeruginosa* vaccines tested so far in humans consisted of antigens targeting single rather than multiple virulence mechanisms—OprF-OprI fusion (13), flagella (14), O antigen-conjugated vaccines (15), high molecular weight alginate (16). Further success in *P. aeruginosa* vaccine development may require a different approach, including bacterial genome evaluation to identify novel antigen combinations potentially addressing multiple virulence mechanisms, such as initial bacterial colonization, immune evasion, colony aggregation and cytotoxicity.

During the past two decades, reverse vaccinology has revolutionized the approach to vaccine research (17, 18), ultimately leading to the development of new generation vaccines based on antigens previously unrecognized by other approaches. One is 4CMenB, the first universal vaccine against serogroup B *Neisseria meningitidis* (MenB), now licensed in several countries worldwide (19, 20). Reverse vaccinology aims at identifying surface exposed proteins, ideally playing a relevant role in pathogenesis, which can serve as targets of the host immune system. This approach has not yet been implemented for *P. aeruginosa*. In this study, reverse vaccinology approach was combined with advanced genomic technologies to select new protein antigens against *P. aeruginosa*. We report that combinations of selected candidates, more than single antigens, effectively control *P. aeruginosa* infections in a mouse model of acute pneumonia.

## Results

### *P. aeruginosa* Antigens Selection by Genome-Wide Screening

**Figure 1:**
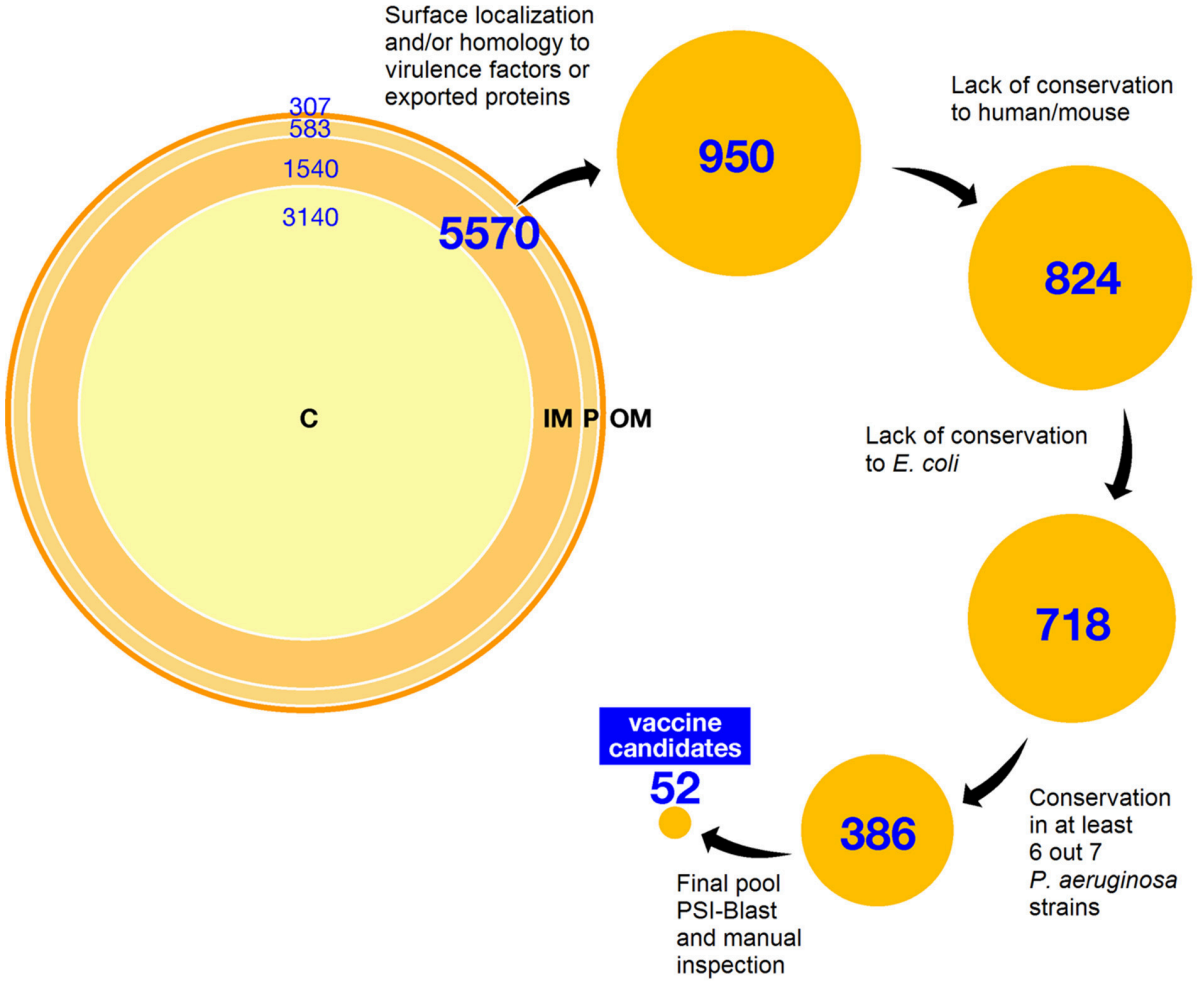
Schematic workflow of antigen selection *in silico*. The complete PAO1 proteome has been analyzed as described in the text. Distribution of ORFs according to predicted sub-cellular localization using Psort is summarized at the top. Below, selection steps succession is reported, indicating the number of candidates (hits) selected after each step. C: cytoplasm; IN: inner membrane; P: periplasm; OM: outer membrane.

Among 5,570 open reading frames (ORFs) encoded by *P. aeruginosa* PAO1 strain (5), we predicted a total of 2,430 surface or membrane-associated proteins by using high throughput bioinformatics localization prediction tools. In particular, subcellular localization was predicted by PsortB software. In the case of predicted non-cytoplasmic polypeptides the presence of signal peptide and localization of cleavage site were predicted with SignalP. N-terminal signatures predictive of lipoproteins were identified by using the LipoP server and putative transmembrane regions were predicted with Tmpred. Among them, 307 were classified as outer membrane proteins or lipoproteins, 583 as periplasmic proteins, and 2,109 as inner membrane proteins. The remaining 2,562 ORFs were predicted to encode cytoplasmic proteins. Since inner membrane proteins are barely exposed on the outside of the bacterium and are difficult to express and purify, all were discarded from selection except those with sequence similarities to known virulence factors or extracellular proteins from other bacterial pathogens. The final selection totaled 950 ORFs (Figure 1).

In a second step of prioritization and to avoid selection of potential self-antigens, we excluded all proteins containing domains with sequence similarity (*E*-value>1e-10) to human and/or mouse proteins, narrowing the selection to 824 proteins. To identify proteins more directly related to *P. aeruginosa* pathogenesis and fitness, and to avoid widespread bacterial housekeeping factors, sequence comparison to *E. coli* K12 whole proteome was performed; we discarded *P. aeruginosa* proteins sharing more than 40% sequence similarity over at least 70% of the length of the *E. coli* counterpart. As sequence conservation is highly desirable for broad-spectrum vaccine candidates, a comparative analysis was performed with the genome sequence of seven published *P. aeruginosa* strains including clinical isolates of different origins (PA14, LESB58, PA7, 2192, C3719, PACS2, RP73); only proteins belonging to the core genome were kept. Candidate selection was also refined by removing short peptides (< 150 aa long), eventually leading to a total of 531 hits. To further prioritize the candidates and reduce the final pool of proteins for experimental testing, a PSI-Blast analysis was conducted; results were manually curated to remove any residual protein putatively involved in intermediate metabolism, DNA synthesis, translation and repair, protein synthesis and transport, and more generally, in any cellular process confined to the bacterial cytoplasm. The final pool of *in silico* selected candidates included 52 antigens—31 proteins of known and 21 of unknown functions (Figure 1 and Table S1). The presence of well-known virulence factors like ExoA and ExoT, as well as relevant outer membrane proteins like OprF and OprH in the final list of candidates confirmed the reliability of the selection strategy.

When tested against an extended panel of 104 *P. aeruginosa* complete genomes, it emerged that all candidates share a mean identity/coverage ratio ranging from 0.78 to 1.00 (Figure S1) confirming that a vast proportion of epitopes potentially presented by each candidate to the immune system is conserved across the natural *P. aeruginosa* population.

### Evaluation of Candidates in a Murine Model of Pneumonia

**Figure 2:**
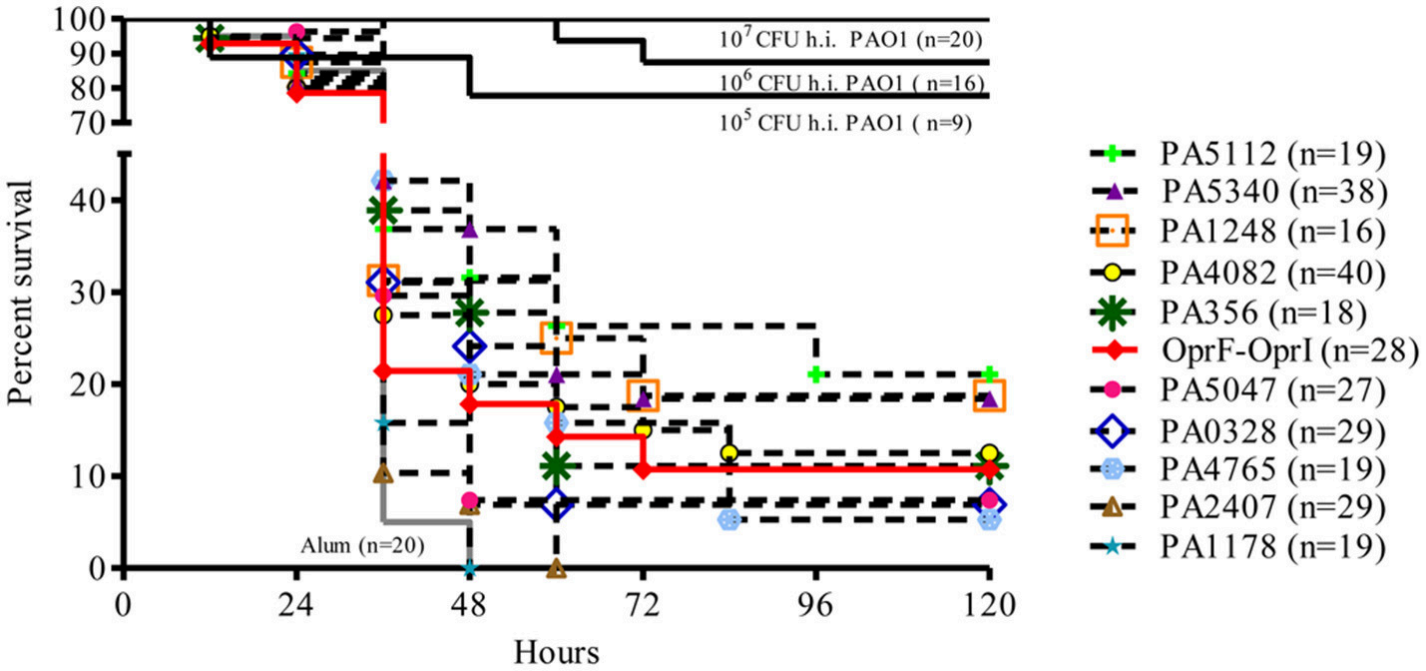
Survival curves of groups of mice immunized with single antigens selected as vaccine candidates. C57Bl/6 male mice were challenged with PAO1 (5^*^10^6^ cfu) 2 weeks after last vaccination with ten single antigens. Comparisons were performed with mice immunized with Alum alone (negative control) and PAO1 heat-inactivated (h.i.) groups (positive control). An additional group was vaccinated with OprF-OprI, tested as clinical vaccine candidate. Data were pooled from at least two/three independent experiments (*n* = 16–40). Results are represented as Kaplan–Meier survival curves and analyzed by the Mantel-Cox test against negative control group. N refers to the number of animals.

Of 52 *P. aeruginosa* vaccine candidates (Table S1), 30 ORFs (57.7%) were successfully expressed in *E. coli* BL21 as His-tag fusions. OprF-OprI fusion, designed according to the known construct used in the recent clinical trial (21), was included in this study. Ability of these antigens to protect against *P. aeruginosa* infection was tested in a mouse model of acute pneumonia (22). C57Bl/6 mice were immunized intraperitoneally (i.p.) with 10 ug of each protein formulated with aluminum hydroxide (Alum) as adjuvant at 0, 21, and 35 days. At day 50, mice were challenged intratracheally (i.t.) with 5 × 10^6^ CFU/lung of *P. aeruginosa* reference strain PAO1 and monitored twice a day for 1 week for health parameters indicative of animal wellness. In this model, all mice immunized with Alum alone (negative control group) consistently showed symptoms of a severe clinical disease and died within 48 h, whereas mice immunized with whole cell inactivated PAO1 bacteria were consistently protected by homologous challenge in a dose-dependent manner. Of the 30 antigens tested, 10 showed a modest increase in survival compared to the negative control (up to 20% at day 5) and were investigated further (Table 1 and Figure 2). The other 20 proteins tested did not differ substantially from the negative control and were discarded (data not shown). Survival of mice vaccinated with OprF-OprI fusion (10.7%) was comparable to that observed by vaccination with the 10 selected proteins.

**Table 1:** Top line vaccine candidates of *P. aeruginosa*.

**Locus_tag**	**Protein annotation**	**Surface exposure^(^^a^^)^**	**Antigenic potential^(^^b^^)^**	**Sequence conservation^(^^c^^)^**	**Sequence conservation^(^^d^^)^**
PA0328	arginine-specific autotransporter AaaA	Yes	Yes	99.3% ± 0.2%	99.2% ± 0.3%
PA1178	outer membrane protein OprH precursor	Yes	Yes	100% ± 0%	99.9% ± 0.2%
PA1248	outer membrane protein AprF precursor	Yes	Yes	99.6%± 0.3%	99.2%± 4.6%
PA2407	putative adhesion protein FpvC	Yes	Yes	100% ± 0%	98.3% ± 10.4%
PA3526	outer membrane protein precursor MotY	Yes	Yes	99.1% ± 0.8%	99.7% ± 0.5%
PA4082	adhesive protein CupB5	Yes	Yes	98.8% ± 0.5%	98.5% ± 2.9%
PA4765	outer membrane lipoprotein OmlA precursor	Yes	Yes	99.7% ± 0.3%	98.9% ± 7.2%
PA5047	putative Zn-dependent protease	Yes	Yes	99.9% ± 0.1%	99.2% ± 7.1%
PA5112	esterase EstA	No	Yes	99.8% ± 0.2%	99.1% ± 7.2%
PA5340	hypothetical protein	Yes	Yes	98.8% ± 0.3%	99.2% ± 0.3%

a *Surface exposure suggested by immunofluorescence microscopy co-localization (see text)*.

b Evaluated by Western Blot against recombinant proteins, P. aeruginosa strain PAO1 and clinical isolate MDR-RP73 (see text). Sequence conservation expressed as mean percentage of amino acid identity ± standard deviation calculated among a collection of CF clinical strains

c *and on the public collection of 104 completed P. aeruginosa genomes available in GenBank (see text)^d^*.

### Evaluation of Combinations of Candidates in a Murine Model of Pneumonia

**Figure 3:**
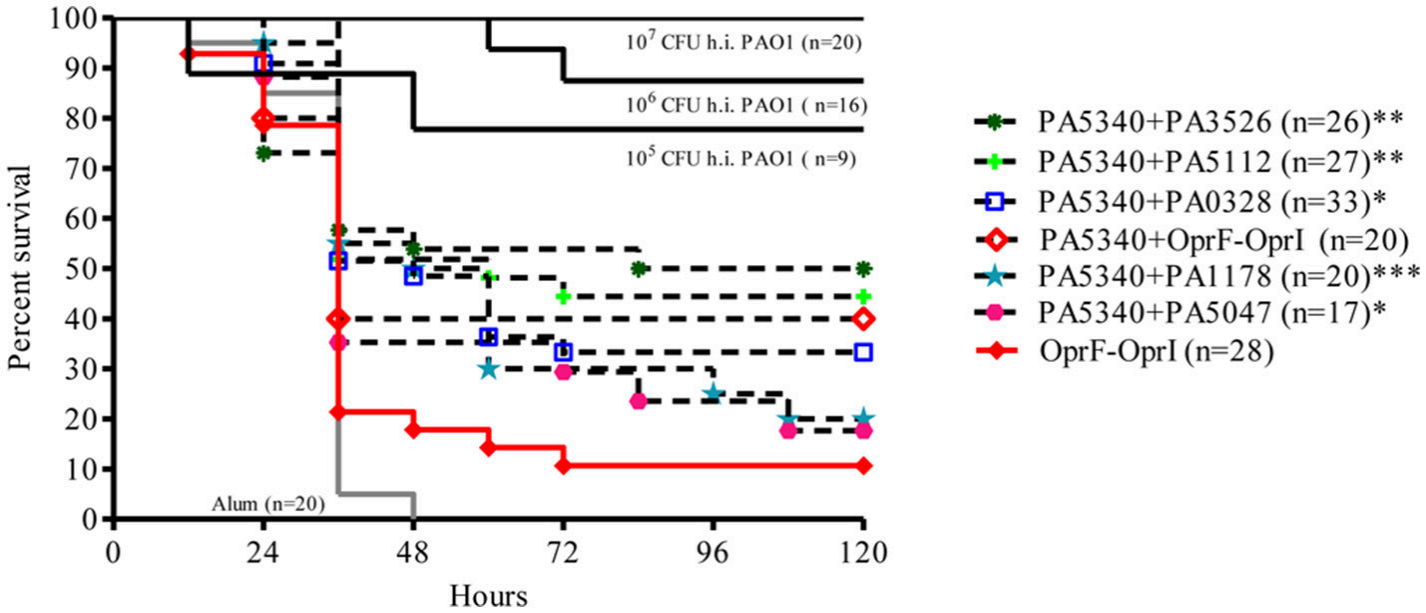
Survival curves of groups of mice immunized with combined antigens selected as vaccine candidates. C57Bl/6 male mice were challenged with PAO1 (5^*^10^6^ cfu) 2 weeks after last vaccination with combined antigens. Comparisons were performed with mice immunized with Alum alone (negative control) and PAO1 heat inactivated (h.i.) groups (positive control). An additional group was vaccinated with OprF-OprI, tested as clinical vaccine candidate. Data were pooled from at least two/three independent experiments (*n* = 17–33). Results are represented as Kaplan–Meier survival curves and analyzed by the Mantel-Cox test against negative control group: *p < 0.05, **p < 0.01, ***p < 0.001. N refers to the number of animals.

In order to increase the survival rate of vaccinated mice, proteins conferring higher protection were pooled in group of two, and 22 combinations were tested. Five combinations, all containing the antigen candidate PA5340, were the most promising (PA5340+PA1178-OprH, PA5340+PA3526-motY, PA5340+PA5112-EstA, PA5340+PA5047, PA5340+PA0328-AaaA). These combinations showed a significant increase in both survival curves (Mantel-Cox test *p* < 0.0002, 0.0019, 0.0027, 0.015, and 0.015, respectively) and mean survival time (one-way ANOVA *p*-value < 0.01) when compared with negative controls (Figure 3 and Figure S2). The best antigens combination was PA5340+PA3526-MotY, with survival increased up to 50%. Three combinations described above, PA5340+PA3526-motY, PA5340+PA5112-EstA, and PA5340+PA0328-AaaA, increased survival significantly when compared with OprF-OprI (Mantel-Cox test 0.0091, 0.0009, and 0.012, respectively). Interestingly, an increase in survival rate was also observed (though not statistically significant) when OprF-OprI was combined with PA5340, going from 10.7% of the fusion alone to 40% when tested in combination (Mantel-Cox test 0.058).

### *In vitro* Characterization of Selected Antigens

**Figure 4:**
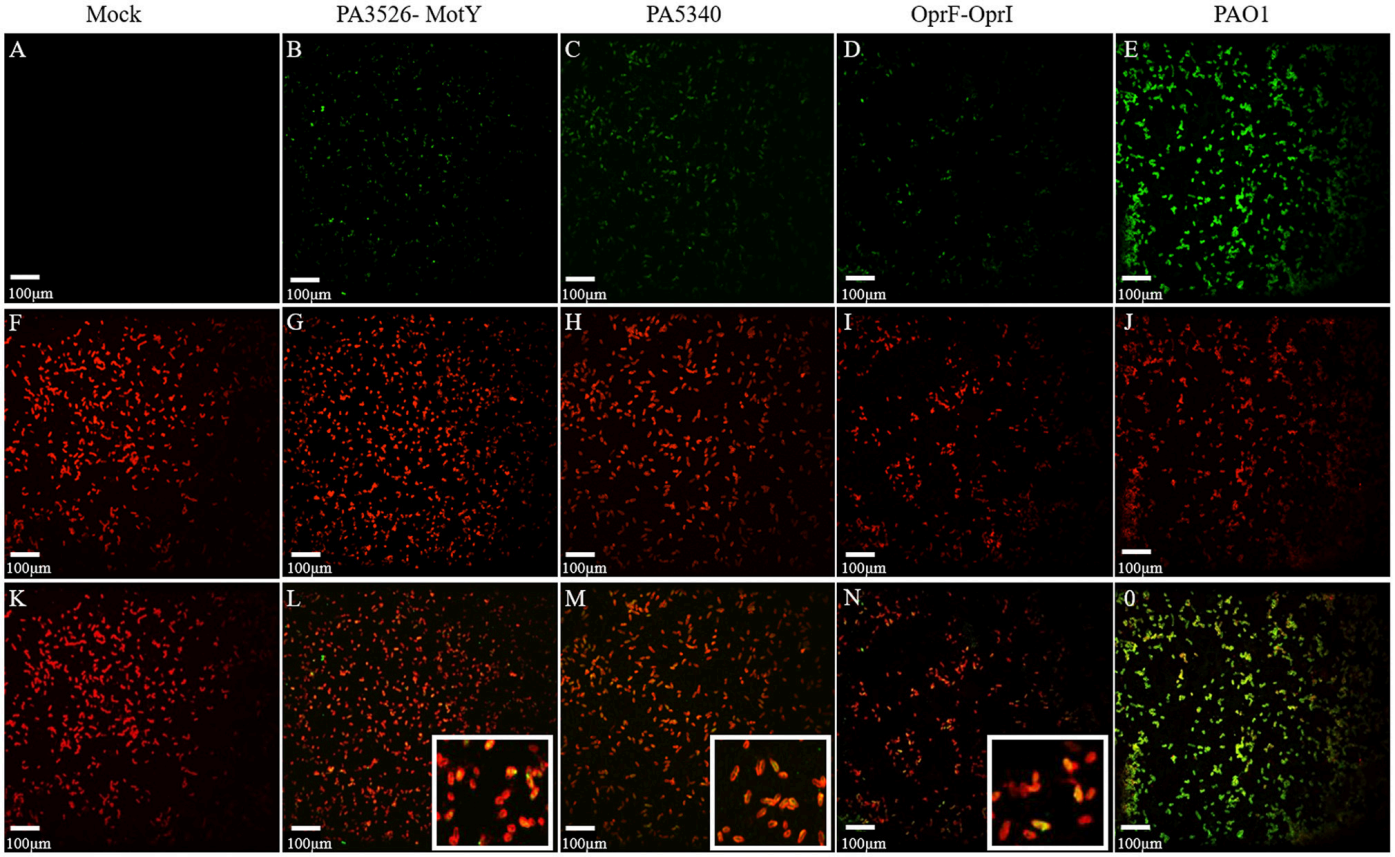
Cellular localization of vaccine candidates PA5340 and PA3526-MotY and controls by immunofluorescence microscopy. Immunofluorescence staining with confocal microscopy shows the localization of antigens (green) **(A–E)** and the PAO1 cell wall (red) **(F–J)**. For antigens localization the antisera of naïve mice **(A)** or immunized with PA3526-MotY **(B)**, PA5340 **(C)**, OprF-OprI **(D)** or heat inactivated PAO1 **(E)** were used. Merged images show the co-localization of the two signals (yellow) **(K–O)** suggesting that proteins could be surface exposed. Detailed co-localization of antigens of interest is shown in the magnification **(L, M, N)**.

To characterize antigenic potential and cellular localization of selected antigens (PA1178-OprH, PA1248-AprF, PA5112-EstA, PA0328-AaaA, PA2407-FpvC, PA3526-MotY, PA4082-CupB5, PA4765-OmlA, PA5047, and PA5340) the antisera obtained immunizing with recombinant proteins were used in Western Blot and immunofluorescence microscopy (Table 1 and Figure 4). All the antisera recognized the recombinant proteins, the homologous *P. aeruginosa* strain PAO1 and the heterologous clinical isolate MDR-RP73; this demonstrates the capacity of the vaccine candidates to induce specific antibody production that can recognize the native proteins.

To determine whether the selected proteins were effectively expressed and exposed on the surface of bacterial cells, a double immunofluorescence was carried out with the murine antisera and a specific antibody for *P. aeruginosa* anti-cell wall as initial characterization. Co-localization of the two signals could suggest that the proteins were present at the bacterial cell surface. As expected, sera of naïve mice did not recognize antigens while antisera of mice immunized with whole cell inactivated PAO1 showed a strong co-localization signal. The same staining was performed with the antisera of mice immunized with the 10 selected antigens and OprF-OprI (Table 1 and Figure 4). According to immunofluorescence microscopy results, all the selected antigens co-localized with the cell surface antibody, with the exception of PA5112-EstA. In particular, a similar co-localization pattern was observed with antisera of PA5340, PA3526-MotY, and OprF-OprI.

### Conservation Profile of Selected Antigens Among a Collection of CF Clinical Isolates

Conservation of the selected antigens among *P. aeruginosa* genomes was initially considered as selection criteria and the candidates were checked in the ensemble of publicly available complete genome sequences. Moreover, we investigated the sequence conservation of the ten most protective antigens in a collection of 19 clinical strains isolated from CF patients at the onset of infection and after years of chronic colonization (Figure S3). Full-length sequences of the ten genes were obtained by PCR from most of the 19 strains. Nucleotide sequences were translated into the amino acid (aa) sequences and compared with PAO1 protein sequence. Overall, identity conservation levels were higher than 98% (Table 1 and Table S2), confirming a strong potential of these proteins to induce effective and cross-protective immunity among *P. aeruginosa* circulating strains.

## Discussion

As an alternative treatment to antibiotics, immunotherapy should represent an option to prevent MDR infections by *P. aeruginosa*. A universal protein-based vaccine against *P. aeruginosa* remains a critical unmet medical need. To identify possible antigens suitable for the development of a *P. aeruginosa* vaccine, we explored the reverse vaccinology approach integrated with additional genomic and bioinformatic approaches. This strategy involved an initial screening of target antigens on the basis of their putative cellular localization; this identified a large number of proteins (2,430 ORFs) predicted to be surface or membrane-associated to various extents. Note that the *P. aeruginosa* genome is larger than most sequenced bacteria (6.3 Mb) (5) and the resulting high number of 5,570 predicted protein encoding genes challenges this approach. Hence, additional criteria were included for antigen selection, with the aim of reducing the pool to a reasonable number of candidates and to rationalize the subsequent experimental steps. In particular, the absence of sequence similarity to proteins encoded by the commensal *E. coli* K12 strain, as well as sequence similarity to proteins involved in primary house-keeping and cell metabolism functions have been used as cutting edge to narrow the shortlist of antigens. Proteins displaying epitopes very similar to those present in widely-conserved proteins from human and mouse origin were also excluded as candidates, as they might be poorly immunogenic and have a high probability of behaving as self-antigens.

Considering that large sequence diversity characterizes *P. aeruginosa* genome, we tested the conservation of selected candidates among the *P. aeruginosa* complete genome sequences available on public databases. This analysis confirmed the presence and conservation of selected antigens in the core genome. Further genomic analysis considered sequence intraclonal diversity of selected antigens in clinical strains isolated from CF patients. It is recognized that *P. aeruginosa* is an antigenically variable microorganism and can undergo phenotypic variation under changing environmental conditions, such as the airways in CF patients (23). In particular, the adaptation process generates unique lineages of *P. aeruginosa* pathogenic variants that differ systematically from environmentally-acquired strains and can escape immune recognition (24). We considered this question worthy of investigation and expanded the comparative sequence analysis on a collection of 19 clinical strains isolated from CF patients at the onset of infection and after years of chronic colonization (25, 26). The selected antigens were conserved among CF clinical isolates indicating that the corresponding genes were not under positive selection and these antigens could be useful for targeting both the initial infecting strains and those promoting progression toward chronic infection. Considering the epidemiology of *P. aeruginosa* infection, both environmental-to-host and patient-to-patient transmission have been described and it appears likely that highly conserved not-adapted antigens might have superior clinical relevance.

Previous studies that tested *P. aeruginosa* antigens provided valuable information on the feasibility of vaccination but were limited by either the number of antigens tested and by redundancy in their selection (10). Abundant surface proteins and oligosaccharides, particular virulence factors have been previously considered. However, none of these started from a large scale screening, performed comparative analysis of the *P. aeruginosa* protein antigens and tested in animal models for preclinical studies. Our screening identified 52 antigens distributed as having known (31) or unknown functions (21). The presence of known virulence factors in the final list of candidates confirmed the reliability of this general selection strategy. We identified a number of proteins, like ExoA and ExoT, as well as relevant outer membrane proteins, like OprF, and OprH, already shown to be required for virulence in *P. aeruginosa*. The outer membrane protein PscC precursor (PA1716) identified in this study was previously identified by integrated genomics and proteomics approach (11). Furthermore, different proteins involved in the chaperone/usher pathways (CupA-E) were identified in this study and Rashid et al. (11). Half of the candidate antigens identified belonged to the functional category of hypothetical, unknown, and unclassified genes (21 genes, 35.8% of the total), suggesting that there is still a large proportion of potentially immunogenic antigens to be discovered within the unexplored part of the *P. aeruginosa*.

Several diverse animal models have been used in preclinical studies of vaccination to evaluate *in vivo* protection against *P. aeruginosa* infection. Mouse models, including burned animals, those with immunocompromised intraperitoneal infection, or with acute pneumonia, have primarily been used in the past. These models were used for preclinical evaluation of candidates like the flagellum, the alginate exopolysaccharide conjugated to tetanus toxoid, polysaccharides, and outer membrane protein such as OprF and OprI. (27–31). Based on our previous experience we consider respiratory infection in immunocompetent mice a highly robust and appropriate model to predict efficacy of vaccine candidates for further clinical testing in patients at risk of respiratory infection, such as VAP or CF (22, 32). The mouse model of acute infection has been extensively employed as the standard in *P. aeruginosa* pathogenesis and efficacy studies (24, 33–35). In this work, C57Bl/6 mice succumbed following infection with high dose of PAO1 virulent *P. aeruginosa* strain. Vaccination with whole cell inactivated *P. aeruginosa* induced an effective immunological response as demonstrated by full protection of the mice. Conversely, mice immunization with the adjuvant alone did not provide any protection as all mice showed severe clinical disease. These data strongly support the choice of this robust mouse model for screening and selecting the best vaccine candidates. Vaccination with the ten purified soluble proteins on our short list demonstrated distinct disease phenotypes, ranging from severe pneumonia to partial protection from a lethal dose of *P. aeruginosa* infection. We report that half of the vaccine candidates screened in this study were more effective when compared to OprF/OprI. It is worth noting that OprF/OprI fusion was one of the treatments evaluated in clinical trial although clinical efficacy has been disappointing (36). Given that vaccines containing several antigens have been shown to confer better protection than those containing only one antigen (20, 37), we decided to assess antigen combinations. Selection of antigens to combine was made from the shortlist of the ten most promising antigens and tested to further increase the vaccine efficacy and survival rate in murine models. This systematic screening identified five combinations, capable of significantly increasing the survival rate among challenged mice. All combinations included PA5340, a hypothetical protein exclusively present in *P. aeruginosa*. The maximum proportion of mice protected against challenge was 50%, achieved with PA5340 combined with PA3526-MotY. These two proteins were capable of triggering a specific immune response and initial characterization could indicate surface exposure. Nevertheless, as their function is still undetermined, it is unlikely that either protein would ever be selected by a traditional approach. Overall this study confirms the capability of reverse vaccinology to give new impetus in the research of vaccines against *P. aeruginosa* infection through the rapid identification of novel vaccine candidates.

## Materials and Methods

### Ethics Statement

Animal studies adhered to the Italian Ministry of Health guidelines for the use and care of experimental animals (protocol #443). The use of the clinical data is in line with study no. 3739 that was approved by the Ethics Commission of Hannover Medical School.

### *In silico* Analysis and Computational Tools

PSORTb (38) was used for the subcellular localization prediction, SingnalP (39) to predict the SPs and their probable cleavage site in secreted proteins, the TatP prediction server to predict the presence of bacterial Tat signal peptides (40), LipoP (41) predict lipoproteins, TMpred to predict transmembrane segments (42).

To check presence and conservation of vaccine candidates in other *P. aeruginosa* genomes, comparative protein sequence analysis against the sequenced genomes was performed using BLAST. The amino acid sequence of strain PAO1 was aligned against the protein translation of complete genome sequences with BLASTp (BLAST 2.2.26+) and against the genome of human and mouse with VAXGEN (http://www.violinet.org/vaxgen/index.php) (43).

### Gene Sequencing

Reference strain PAO1 (5) and 19 *P. aeruginosa* clinical strains isolated from CF patients attending the Medizinische Hochschule Hannover and described previously (25, 26) were used to sequence the genes of vaccine candidates. PCR genes amplification was carried out using the list of primers detailed in the Supplementary Information.

### Cloning, Expression, and Purification of *P. aeruginosa* Recombinant Proteins

Polypeptides antigens from *P. aeruginosa* PAO1 were PCR-amplified using specific oligonucleotides and *P. aeruginosa* chromosomal DNA as templates. Resulting PCR products were cloned in pET15b (Novagen) using the PIPE method (44). To express cloned proteins, BL21(DE3)T1^r^ clones containing pET15b constructs were grown in LB medium containing 100 μg/ml Ampicilin at 37°C until OD_600_ = 0.5. Protein expression was induced by adding 1 mM IPTG and growing at the same temperature for additional 3 hrs. Conventional protein extractions and SDS-Page were performed to check protein expression. Western Blot was used to confirm proper expression of tested *P. aeruginosa* antigens.

Protein purification has been performed as reported previously (45). Briefly, bacteria cells undergone to mechanical or chemical lysis and recombinant polypeptides were recovered from crude cell extracts by immobilized-metal ion affinity chromatography (IMAC) using His MultiTrap™ HP 50 mL NiSepharose High-Performance 96 well-vacuum plates (GE Healthcare). Polypeptides expressed as insoluble inclusion bodies were solubilized in 50 mM Tris–HCl elution buffer, pH 8.8 containing 8 MUrea, 1 mM TCEP-HCl, and 250 mM imidazole. Renaturation was performed by dialysis in 50 mM NaH2PO4, pH 8.8 containing 10% (v/v) glycerol, 0.5 M arginine, 5.0 mM of reduced glutathione, 0.5 mM of oxidized glutathione in the presence of 4, 2, or 0 M urea. Protein concentration was determined using the Micro BCA protein assay reagent kit (Pierce). Protein purity was checked by SDS-PAGE CRITERION XT Precast Gel (Biorad) followed by Coomassie blue staining.

### Mouse Immunizations and Protection Model

C57BL/6NCrlBR 5 week-old male mice (Charles River) were immunized i.p. at day 0, 21, and 35 with recombinant proteins formulated with Alum, either individually or as a combination of proteins. The formulations were optimized for pH and osmolarity. Each antigen was used at 10 μg/formulation/animal. The final concentration of Alum was 2 mg/ml in 10 mM histidine buffer (pH 6.5). Negative control mice were immunized with Alum alone, while positive control mice were boosted with whole cell heat-inactivated PAO1 strain at different doses (10^5^-10^7^ CFU). To obtain the antisera, mice were bled at day - 1, 34, and 49. At day 50, mice were challenged with 5 × 10^6^ CFU of *P. aeruginosa* PAO1 strain by acute infection and monitored every 12 h for general wellness as detailed in the Supplementary Information.

### Western Blot and Immunofluorescence Microscopy

Specific antisera from immunized mice were used to confirm protein expression by Western Blot and surface localization of antigens by immunofluorescence as detailed in the Supplementary Information.

### Statistical Analysis

Statistical calculations and tests were performed using Mantel-Cox test and one-way ANOVA, considering *p* < 0.05 as the limit of statistical significance.

## Author Contributions

AB and VM: conceiving and designing the experiments. BA-F, IB, MS, SB, SM, AC, and MD: performing experiments. AB, BA-F, IB, MS, and VM: analyzing data and interpretation of the experiments results. AB, BA-F, IB, MS, and VM: writing the manuscript.

### Conflict of Interest Statement

MS, SM, SB, AC, and VM were employees of Novartis Vaccines and Diagnostics Srl at the time of the study (now part of the GSK group of companies). They are now employees of the GSK group of companies. MD is a student at the University of Torino and participated in a post graduate studentship program at GSK. The remaining authors declare that the research was conducted in the absence of any commercial or financial relationships that could be construed as a potential conflict of interest.
