# Supplementary material for: Genome-Based Approach Delivers Vaccine Candidates Against Pseudomonas aeruginosa
Source: Front Immunol. 2019 Jan 9;9:3021. doi: 10.3389/fimmu.2018.03021 (PMC6334337; doi:10.3389/fimmu.2018.03021)
Supplement: Supplementary file 1 [file Data_Sheet_1.pdf]

Genome-based approach delivers vaccine candidates against *Pseudomonas aeruginosa*.

Irene Bianconi [1§], Beatriz Alcalá-Franco [1§], Maria Scarselli [2§], Mattia Dalsass [2, 3], Scilla Buccato [2], Annalisa Colaprico [2], Sara Marchi [2], Vega Masignani [2*], Alessandra Bragonzi [1*]

[1] Infection and Cystic Fibrosis Unit, Division of Immunology, Transplantation and Infectious Diseases, IRCCS San Raffaele Scientific Institute, Milano, Italy

[2] GSK, Siena Italy

[3] Dipartimento di Scienze Cliniche e Biologiche, Università degli Studi di Torino, Torino, Italy

[§]These authors contributed equally to the work and share first authorship.

*These authors have equal contributions and share senior authorship.

Supplementary Information.

Evaluation of the virulence role of selected vaccine candidates

PA0328 arginine-specific autotransporter AaaA

AaaA plays a key role in the establishment of the infections as it releases arginine (from the aminoterminus of di and tripeptides) that can be used as a nutrient for growth (1) and it is able to promote chronic skin wound infections in murine models (2).

PA1178 outer membrane protein OprH precursor

The function of OprH is to stabilise the outer membrane (OM) (3), and it is under the regulation of the PhoP-PhoQ system (4), and it has been identified as a component of the Outer Membrane Vescicoles (OMVs) produced by PAO1 both in planktonic and sessile lifestyle (5) (6). The OprH-PhoP-PhoQ system, induced upon Mg2+ starvation, is involved in the resistance to the polycationic antibiotic polymyxin B (7).

PA1248 outer membrane protein AprF precursor

AprF is a secreted proteases and virulence determinant induced by the AlgU-dependent conversion to mucoidy (8) (9).

PA2407 FpvC

The periplasmic protein FpvC, belonging to the FpvCDEF operon (an ABC transporter involved in pyoverdine-Fe uptake), acts as a Fe2+ chelator (10).

Defects in the operon affect neither the growth nor pyoverdine production under iron-limited environment conditions in PAO1 (11). However, the deletion of the complex formed by fpvWXYZCDEF in PAO1 results in defective growth, increased H2O2 sensitivity and decreased virulence (12).

PA3526 outer membrane protein precursor MotY

MotY is a probable OM protein involved in flagellar motility (13). The deletion of motY in PAO1 or PAK backgrounds severely affects swimming motility in semisolid motility plates (14). MotY is required for the proper flagellar function, mutations in motY combined with others in motAB and motCD dramatically reduce the swimming motility or render the cells nonmotile (14).

PA4082 adhesive protein CupB5

CupB5 nonchaperone-usher gene product involved in the assembling adhesive pili on the bacterial surface (14).

Overexpression of CupB5, in the absence of its chaperone, promotes the conversion to a mucoid phenotype through the AlgU stress-response pathway and a CupB5 peptide plays a key role in the cleavage of MucA by AlgW (15).

PA4765 outer membrane lipoprotein OmlA precursor

Even if the precise function is unclear, OmlA is putative involved in (i) maintaining the cell envelope integrity and (ii) antibiotic susceptibility: an OmlA mutant showed increased susceptibility to several antibiotics (including nalidixic acid, rifampin, novobiocin, and chloramphenicol) belonging to different classes and it was found to be hypersusceptible to anionic detergents (16).

PA5112 esterase EstA

EstA is an autotransporter protein present in the OM and OMVs (17).

Inactivation of the estA gene results in rhamnolipid deficiency as well as defect in motility and biofilm formation (18). EstA is able to induce nitric oxide and pro-inflammatory cytokines in macrophages (19) and it is required for full virulence in a rat model of chronic lung infection (20).

METHODS

Gene sequencing and analysis. PCR genes amplification was carried out using the following list of primers: PA1178: For-TAGAAAGCCTAGACCCTACTTG, Rev-TCTTCCACTACCAGCAGTTT; PA1248: For- CTGCTCAATTACCTGTTCAAG, Rev- AAGACAAACTACCGAAGACACT; PA5112: For-CTGATCGAGCGCGACAATAC, Rev-GTGTCGTCCTCGTACTCACG; pa0328: For-ATGAAACGGTCCGCATCCTG Rev-CGAGCCGAACCTGTTCTACGT; PA2407: For- CATTGATCGCACATCGACTC, Rev-GTCTTGACCAGCGAACTCTTG; PA3526: For-GATACATCCTTCGTATTTGGAC, Rev- CATTCGGGAAATTACAGAGG; PA4082: For- CAGAGAGATACCCGTAGGAGTT, Rev-CTCATGGAACTCTCCAAGATT; PA4765: For-

GAAGTTCGCTATTTTCAACCAT, Rev- GTATCTTCGGCAAGCTCCTG; PA5047: For-GTATCTGGCTGGAGATGGAC, rev-TTTATTGCTTGTTGGACAGAC; PA5340: For- CTCGAGTAAGCCGGATGTTC, Rev- GCGGACTGTACTTCCTCTGG.

The amplified DNA samples were sequenced by standard automated DNA sequence technology. The sequence results were compared within *P. aeruginosa* clonal lineages with BioEdit v7.0.5 (http://www.mbio.ncsu.edu/BioEdit/bioedit.html) to determine the occurrence of sequence variants within the selected genes and identify the amino acids.

Mouse model. Mice were kept in pathogen-free conditions and tested as previously described (21-24). For infection experiments, mice were anesthetized by intraperitoneal injection of a solution of Avertin (2,2,2-tribromethanol, 97%) in 0.9% NaCl, at a volume of 0.015 ml/g body weight. The mice were positioned supine. The trachea was directly visualized by ventral midline, exposed and intubated with a sterile, flexible 22-g cannula attached to a 1 ml syringe. An inoculum of 60 μl of bacterial suspension was implanted in the lung via the cannula. After inoculation, all incisions were sutured. All mice were kept under specific pathogen-free conditions in sterile cages placed in a ventilated isolator. Fluorescent lights were cycled 12h on/12h off; ambient temperature (23±1°C) and relative humidity (40-60%) were regulated. The mice were fed with standard rodent autoclaved chow and autoclaved tap water.

The mice were monitored twice-a-day for scruffy coat, inactivity, loss of appetite, poor locomotion and painful posture.

Western Blot (WB). Recombinant proteins were solubilized in sample buffer and separated by 12% sodium dodecyl sulphate (SDS)–PAGE. Proteins were electro-transferred onto nitrocellulose membranes (0.45μm) using a mini trans blotter (Bio-Rad) according to the manufacturer's instructions. The membranes were blocked 1 hour at RT in blocking buffer. Pools of sera from each group of immunized mice, collected at day 49, were incubated O/N at 4°C. Specific antibodies were detected with polyclonal rabbit anti-mouse (Dako). Whole cell lysates of the homologous strain PAO1 and the clinical isolate RP73, both in stationary and exponential growth phases, were separated by SDS-PAGE, and WB was performed as described.

Immunofluorescence microscopy. Protein localization was performed on PAO1

growth spread on a coated slide. After fixation with 4% PFA, samples were washed three times with 0,1% tween20 in PBS. Bacteria incubation in blocking buffer (3% BSA 10% swine serum in PBS)was followed by incubation with primary antibodies, mouse anti sera (1:50) and specific rabbit anti *P. aeruginosa* cell wall antibodies (1:800), kindly provided by Dr. Gerald Pier, in 0,1% tween20 in PBS. Primary antibodies were labelled with anti-mouse Fab-Jackson (1:200) and Texas Red-labelled goat anti-rabbit (1:2000). Next, samples were washed with PBS and Daco Fluorescent Mounting Medium was use to cover the slides. Immunofluorescence images were recorded with an EM-CCD Hamamatsu C9100 camera (Hamamatsu Photonics, Hamamatsu City, Japan) mounted on an UltraVIEW Spinning Disk Confocal Microscope (Perkin Elmer, Waltham, MA, USA).

Virulence factors screening. The shortlisted 52 vaccine candidates were compared with the *P. aeruginosa* virulence factors present in the Virulence Factor Database (VFDB) (25) in order to identify the antigens with a known role in the pathogenesis of the bacterium.

Table S1. Top line vaccine candidates of *P. aeruginosa*.
Subcellular localization was predicted with Psot server (http://psort1.hgc.jp/form.html). [(a)] OM = outer membrane; IM =inner membrane; P=periplasmic; C = cytoplasmic. [(b)]Comparison with the virulence factors present in Virulence Factors Database (VFDB).

Locus_ tag	Protein annotation	length (aa)	Predicted localization [(a)]	Role in virulence[(b)]	Expression
**PA0041**	hemagglutinin	3535	OM		no
**PA0044**	exoenzyme T (exoT)	457	C	TTSS translocated effectors	no
**PA0077**	IcmF1	1101	IM	Hcp secretion island-1 encoded type VI secretion system (H-T6SS)	no
**PA0126**	hypothetical protein	206	OM		yes
**PA0151**	putative TonB-dependent receptor	795	OM		no
**PA0189**	putative porin	452	OM		yes
**PA0274**	hypothetical protein	256	P		yes
**PA0328**	hypothetical protein	647	OM		yes
**PA0537**	hypothetical protein	202	OM		yes
**PA0595**	organic solvent tolerance OstA precursor	924	OM		yes
**PA0690**	hypothetical protein	4180	IM		no
**PA0692**	hypothetical protein	544	OM		no
**PA0696**	hypothetical protein	568	OM		no
**PA0737**	hypothetical protein	151	OM		yes
**PA0958**	outer membrane porin OprD precursor	443	OM		no
**PA1011**	outer membrane protein assembly	396	OM		no

	BamC precursor				
**PA1048**	probable outer membrane protein	293	OM		no
**PA1086**	flagellar hook-associated protein FlgK	683	P	Flagella	yes
**PA1106**	hypothetical protein	237	OM		yes
**PA1148**	exotoxin A precursor	638	P	Exototoxin-A (ETA)	no
**PA1178**	outer membrane protein OprH precursor	200	OM		yes
**PA1248**	outer membrane protein AprF precursor	481	P		yes
**PA1324**	hypothetical protein	170	OM, lipoprotein		yes
**PA1578**	hypothetical protein	251	P		no
**PA1716**	outer membrane protein PscC precursor	600	P	TTSS	no
**PA1777**	outer membrane porin OprF precursor	350	OM		yes
**PA1812**	membrane-bound lytic murein transglycosylase D precursor	534	OM		no
**PA1952**	hypothetical protein	250	OM		no
**PA1954**	hypothetical protein	340	OM		yes
**PA2130**	usher CupA3 protein	872	OM		no
**PA2407**	putative adhesion protein FpvC	317	P		yes
**PA2793**	hypothetical protein	344	OM, lipoprotein		yes
**PA3115**	motility protein FimV	919	OM	Type IV pili biosynthesis	no

**PA3239**	hypothetical protein	267	OM		no
**PA3526**	outer membrane protein precursor	321	P	Flagella	yes
**PA3535**	putative serine protease	995	OM		yes
**PA3647**	periplasmic chaterone HlpA	168	P		yes
**PA3648**	outer membrane protein Opr86	797	OM		no
**PA3692**	OmpA family protein	261	OM , lipoprotein		yes
**PA4058**	hypothetical protein	156	OM		no
**PA4082**	adhesive protein CupB5	1018	IM		yes
**PA4221**	Fe(III)-pyochelin outer membrane receptor precursor	720	OM	Pyochelin receptor	no
**PA4370**	metalloproteinase outer membrane protein precursor	446	OM		yes
**PA4578**	hypothetical protein	162	OM		yes
**PA4667**	hypothetical protein	590	OM		yes
**PA4710**	heme/hemeoglobin uptake outer membrane receptor PhuR precursor	764	OM		yes
**PA4735**	OmpA family protein	1088	OM		yes
**PA4765**	Outer membrane lipoprotein OmlA precursor	176	OM		yes

**PA5047**	putative Zn-dependent protease	479	OM, lipoprotein	yes
**PA5112**	esterase EstA	646	OM	yes
**PA5340**	hypothetical protein	243	OM, lipoprotein	yes
**PA5441**	hypothetical protein	733	P	no

Table S2: Amino acid substitution between PAO1 and a collection of *P. aeruginosa* clinical isolates.

[a] Amino acid present in the reference strain PAO1 (and position of the amino acid) – amino acid substitution clinical isolates specified in [b].

Recombinant proteins	aa Mutation [a]	*P. aeruginosa* clinical isolates [b]
PA1178	-	
PA1248	Q_{53}-M	AA2, AA43, AA44
	P_{450}-Q	
	P_{238}-A	MF1, MF51
	T_{82}-A	SG1, SG57, AA2, AA43, AA44
	F_{88}-L	TR1, TR66, TR67, KK1, KK71, KK72, BST2, BST44
	S_{478}-G	AA2, AA43, AA44, MF1, MF51
PA5112	A_{641}-V	MF1, MF51
	N_{437}-D	TR1, TR66, TR67, KK1, KK71, BST2
	T_{534}-S	AA2, AA43, AA44, TR1, TR66, TR67 MF1, MF51, KK1, KK71, BST2
PA0328	N_{195}-S	AA2, AA43, AA44, BT2, BT73, AA2, AA43, AA44, BST2, BST44, KK1, MF1, MF51, TR1, TR66, TR67
	K_{55}-E	SG1, SG57, SG58, BT2, BT73, AA2, AA43, AA44, BST2, BST44, KK1, MF1, MF51, TR1, TR66, TR67
	Q_{314}-L	
	H_{516}-R	SG1, SG57, SG58, BT2, BT73
	D_{580}-E	
	L_{609}-I	
	T_{585}-I	BST2, BST44, KK1
	R_{416}-S	BST44
	A_{486}-T	
	D_{538}-E	
	V_{600}-E	
PA2407	-	
PA3526	D_{169}-E	BT72, BT73
	S_{111}-N	TR1, TR66, TR67
	S_{241}-N	MF51
PA4082	S_{23}-C	SG1, SG57, SG58
	N_{293}-S	
	V_{367}-L	
	N_{748}-Y	

	E_{964}-K	
	H_{992}-R	BT2, BT72, BT73
	N_{748}-D	BT72
	N_{748}-Y	BT73
	N_{187}-Q	KK1
	S_{280}-T<br>K_{292}-S	MF1
	H_{187}-Q	MF1, BST 44
	N_{318}-K	BT2, BT72, BT73, TR1, TR67
	V_{39}-A	SG1, SG57, SG58, BT2, BT72, BT73, TR1, TR67,
	S_{629}-N<br>A_{633}-T<br>G_{643}-A<br>G_{700}-A<br>M_{701}-T	SG1, SG57, SG58, BT2, BT72, BT73, KK1, BST44
	T_{801}-M	SG1, SG57, SG58, BT2,BT72,BT73, KK1
	S_{818}-G<br>V_{878}-F<br>N_{915}-S	SG1, SG57, SG58, BT2, BT72, BT73
	A_{882}-T<br>G_{916}-S	TR1, TR67, MF1
	S_{917}-G	SG1, SG57, SG58, BT2, BT72, BT73, TR1, TR67, MF1
	N_{403}-S<br>L_{958}-V	SG1, SG57,SG58, BT2, BT71, BT72, TR1, TR67, MF1, KK1, BST44
PA4765	A_{138}-E	SG1, SG57, SG58, BT2, BT72, BT73, MF1, MF51
PA5047	A_{421}-T	MF1, MF51
PA5340	S_{142}-C	SG1, SG57,SG58
	T_{12}-A	BT2, BT72, BT73
	E_{104}-C	TR1, TR66, TR67
	S_{110}-N	MF1, MF51
	E_{114}-D	AA2, AA43, AA44, KK1, KK71, KK72
	S_{128}-N	SG1, SG57,SG58, BT2, BT71, AA2, AA43, AA44, TR1, TR66, TR67, MF1,MF51, KK1, KK71, KK72, BST2

REFERENCES

1. Das P, Lahiri A, Lahiri A, Chakravortty D. *Modulation of the arginase pathway in the context of microbial pathogenesis: A metabolic enzyme moonlighting as an immune modulator*. PLoS Pathog (2010) doi:10.1371/journal.ppat.1000899

2. Luckett JCA, Darch O, Watters C, AbuOun M, Wright V, Paredes-Osses E, Ward J, Goto H, Heeb S, Pommier S, et al. *A Novel Virulence Strategy for Pseudomonas aeruginosa Mediated by an Autotransporter with Arginine-Specific Aminopeptidase Activity*. PLoS Pathog (2012) **8**: doi:10.1371/journal.ppat.1002854

3. Chevalier S, Bouffartigues E, Bodilis J, Maillot O, Lesouhaitier O, Feuilloley MGJ, Orange N, Dufour A, Cornelis P. *Structure, function and regulation of Pseudomonas aeruginosa porins*. FEMS Microbiol Rev (2017) doi:10.1093/femsre/fux020

4. Edrington TC, Kintz E, Goldberg JB, Tamm LK. *Structural basis for the interaction of lipopolysaccharide with outer membrane protein H (OprH) from Pseudomonas aeruginosa*. J Biol Chem (2011) doi:10.1074/jbc.M111.280933

5. Choi D-S, Kim D-K, Choi SJ, Lee J, Choi J, Rho S, Park S-H, Kim Y-K, Hwang D, Gho YS. *Proteomic analysis of outer membrane vesicles derived from Pseudomonas aeruginosa*. Proteomics (2011) **11**:3424–3429. doi:10.1002/pmic.201000212

6. Couto N, Schooling SR, Dutcher JR, Barber J. *Proteome Profiles of Outer Membrane Vesicles and Extracellular Matrix of Pseudomonas aeruginosa Biofilms*. J Proteome Res (2015) doi:10.1021/acs.jproteome.5b00312

7. Olaitan AO, Morand S, Rolain JM. *Mechanisms of polymyxin resistance: Acquired and intrinsic resistance in bacteria*. Front Microbiol (2014) doi:10.3389/fmicb.2014.00643

8. Duong F, Lazdunski A, Carni B, Murgier M. *Sequence of a cluster of genes controlling synthesis and secretion of alkaline protease in Pseudomonas aeruginosa: relationships to other secretory pathways*. Gene (1992) doi:10.1016/0378-1119(92)90160-Q

9. Firoved AM, Deretic V. *Microarray analysis of global gene expression in mucoid Pseudomonas aeruginosa*. J Bacteriol (2003) 185:1071–1081. doi:10.1128/JB.185.3.1071-1081.2003

10. Ganne G, Brillet K, Basta B, Roche B, Hoegy F, Gasser V, Schalk IJ. *Iron Release from the Siderophore Pyoverdine in Pseudomonas aeruginosa Involves Three New Actors: FpvC, FpvG, and FpvH*. ACS Chem Biol (2017) doi:10.1021/acschembio.6b01077

11. Brillet K, Ruffenach F, Adams H, Journet L, Gasser V, Hoegy F, Guillon L, Hannauer M, Page A, Schalk IJ. *An ABC transporter with two periplasmic binding proteins involved in iron acquisition in pseudomonas aeruginosa*. ACS Chem Biol (2012) doi:10.1021/cb300330v

12. Gao L, Guo Z, Wang Y, Wang Y, Wang K, Li B, Shen L. *The Two-Operon-Coded ABC Transporter Complex FpvWXYZCDEF is Required for Pseudomonas aeruginosa Growth and Virulence Under Iron-Limiting Conditions*. J Membr Biol (2018) doi:10.1007/s00232-017-9979-5

13. McCarter LL. *MotY, a component of the sodium-type flagellar motor*. J Bacteriol (1994) doi:10.1128/jb.176.14.4219-4225.1994

14. Doyle TB, Hawkins AC, McCarter LL. *The complex flagellar torque generator of Pseudomonas aeruginosa*. J Bacteriol (2004) doi:10.1128/JB.186.19.6341-6350.2004

15. de Regt AK, Yin Y, Withers TR, Wang X, Baker TA, Sauer RT, Yu HD. *Overexpression of CupB5 activates alginate overproduction in Pseudomonas aeruginosa by a novel AlgW-dependent mechanism*. Mol Microbiol (2014) doi:10.1111/mmi.12665

16. Ochsner UA, Vasil AI, Johnson Z, Vasil ML. *Pseudomonas aeruginosa fur overlaps with a gene encoding a novel outer membrane lipoprotein, omlA*. J Bacteriol (1999)

17. Wilhelm S, Tommassen J, Jaeger KE. *A novel lipolytic enzyme located in the outer membrane of Pseudomonas aeruginosa.* J Bacteriol (1999) doi:DOI 10.1002/jrs.1250200712

18. Wilhelm S, Gdynia A, Tielen P, Rosenau F, Jaeger KE. The autotransporter esterase EstA of *Pseudomonas aeruginosa is required for rhamnolipid production, cell motility, and biofilm formation*. J Bacteriol (2007) doi:10.1128/JB.00023-07

19. Kang EH, Gebru E, Kim MH, Cheng H, Park SC. *EstA protein, a novel virulence factor of Streptococcus pneumoniae, induces nitric oxide and pro-inflammatory cytokine production in RAW 264.7 macrophages through NF-κB/MAPK.* Microb Pathog ***(2009)*** doi:10.1016/j.micpath.2009.07.002

20. Potvin E, Lehoux DE, Kukavica-Ibrulj I, Richard KL, Sanschagrin F, Lau GW, Levesque RC. *In vivo functional genomics of Pseudomonas aeruginosa for high-throughput screening of new virulence factors and antibacterial targets*. Environ Microbiol (2003) doi:10.1046/j.1462-2920.2003.00542.x

21. Williams, B.J., J. Dehnbostel, and T.S. Blackwell, *Pseudomonas aeruginosa: host defence in lung diseases*. Respirology, (2010) **15**(7): p. 1037-56.

22. Gellatly, S.L. and R.E. Hancock, *Pseudomonas aeruginosa: new insights into pathogenesis and host defenses*. Pathog Dis (2013) **67** (3): p. 159-73.

23. Stover, C.K., Pham XQ, Erwin AL, Mizoguchi SD, Warrener P, Hickey MJ, Brinkman FS, Hufnagle WO, Kowalik DJ, Lagrou M, Garber RL, Goltry L, Tolentino E, Westbrock-Wadman S, Yuan Y, Brody LL, Coulter SN, Folger KR, Kas A, Larbig K, Lim R, Smith K, Spencer D, Wong GK, Wu Z, Paulsen IT, Reizer J, Saier MH, Hancock RE, Lory S, Olson MV., *Complete genome sequence of Pseudomonas aeruginosa PA01, an opportunistic pathogen*. Nature (2000) **406**(6799): p. 959-64.

24. Chastre, J. and J.Y. Fagon, Ventilator-associated pneumonia. Am J Respir Crit Care Med (2002) **165**(7): p. 867-903.

25. Chen, L., Xiong, Z., Sun, L., Yang, J., and Jin, Q., *VFDB 2012 update: Toward the genetic diversity and molecular evolution of bacterial virulence factors.* Nucleic Acids Res. (2012) **40** (Database issue):D641-5.
