# Supplementary figures and images for: Genome-Based Approach Delivers Vaccine Candidates Against Pseudomonas aeruginosa
Source: Front Immunol. 2019 Jan 9;9:3021. doi: 10.3389/fimmu.2018.03021 (PMC6334337; doi:10.3389/fimmu.2018.03021)

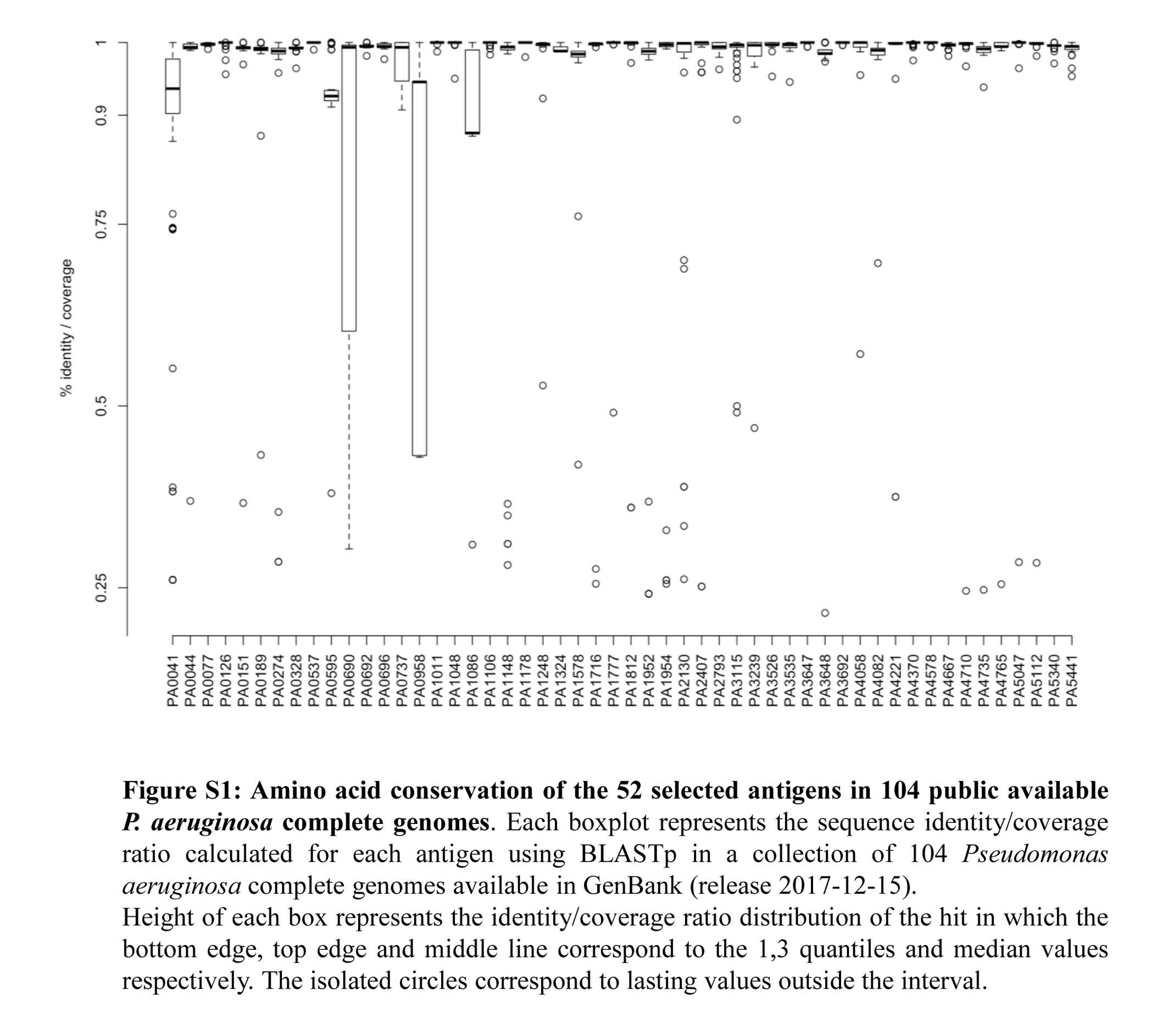

Supplement: Supplementary file 2 [file Image_1.TIF]

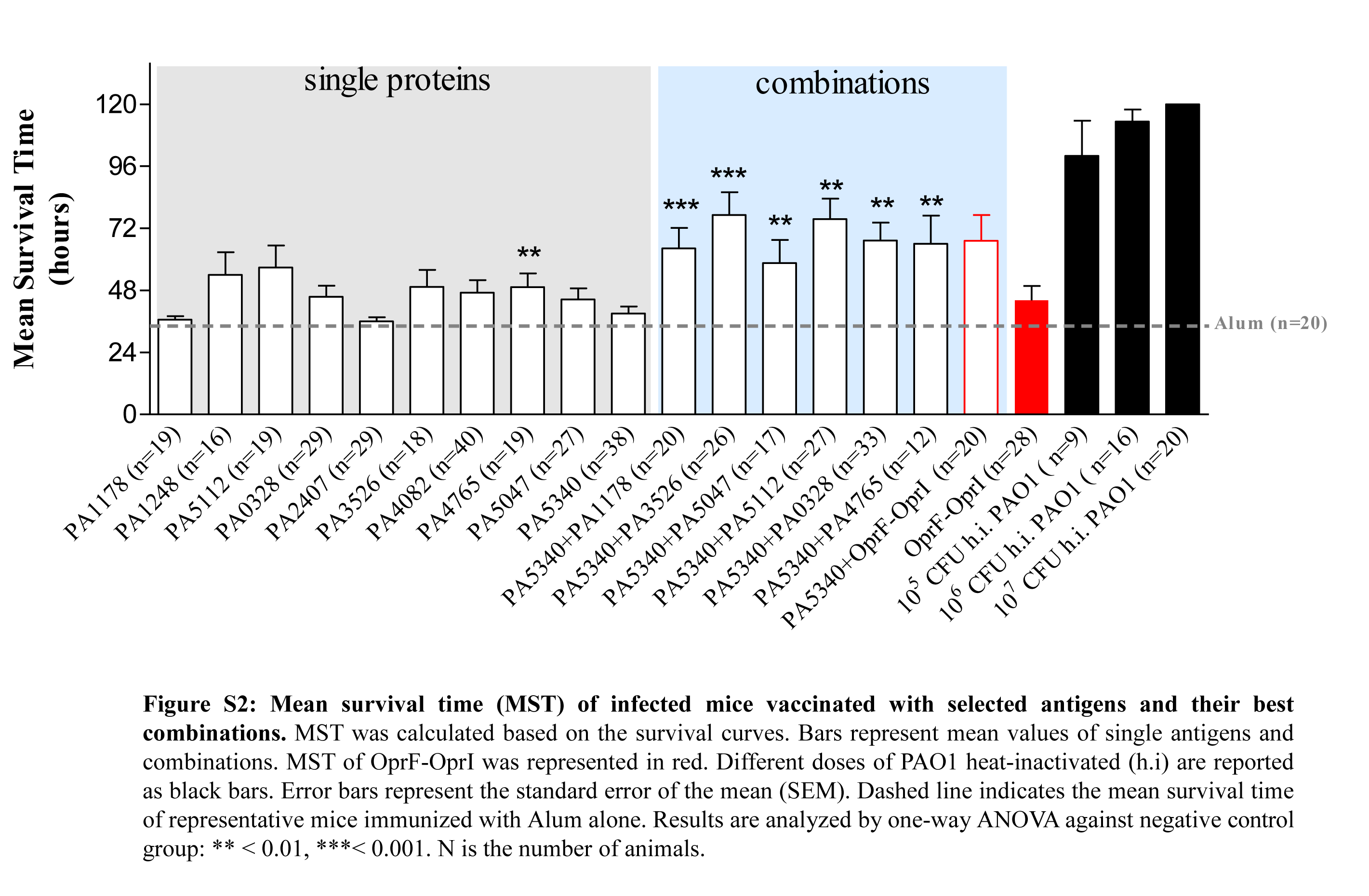

Supplement: Supplementary file 3 [file Image_2.tif]

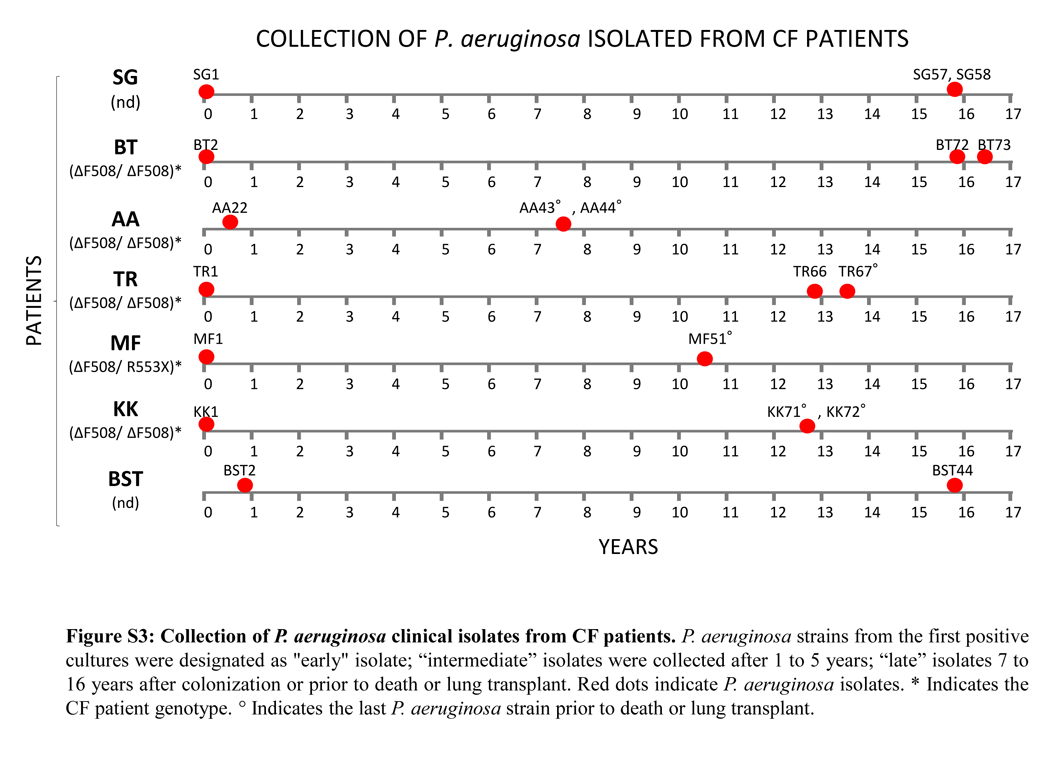

Supplement: Supplementary file 4 [file Image_3.TIF]
